# Vaccination coverage against human papillomavirus (HPV) and associated factors in female academics from a university in southwestern Goiás, Brazil

**DOI:** 10.11606/s1518-8787.2021055003144

**Published:** 2021-10-13

**Authors:** Paulo Sérgio de Oliveira, Carla Vitola Gonçalves, Guilherme Watte, Juvenal Soares Dias da Costa

**Affiliations:** I Universidade de Rio Verde Faculdade de Medicina Rio VerdeGO Brasil Universidade de Rio Verde. Faculdade de Medicina. Rio Verde, GO, Brasil; II Universidade Federal do Rio Grande Faculdade de Medicina Rio GrandeRS Brasil Universidade Federal do Rio Grande. Faculdade de Medicina. Rio Grande, RS, Brasil; III Universidade Católica do Rio Grande do Sul Programa de Pós-Graduação em Medicina e Ciências da Saúde da Pontifícia Porto AlegreRS Brasil Universidade Católica do Rio Grande do Sul. Programa de Pós-Graduação em Medicina e Ciências da Saúde da Pontifícia, Porto Alegre, RS, Brasil; IV Universidade do Vale do Rio dos Sinos Programa de Pós-Graduação em Saúde Coletiva São LeopoldoRS Brasil Universidade do Vale do Rio dos Sinos. Programa de Pós-Graduação em Saúde Coletiva. São Leopoldo, RS, Brasil

**Keywords:** Young Adult, Students, Health Occupations, Papillomavirus Infections, prevention & control, Papillomavirus Vaccines, supply & distribution, Vaccination Coverage

## Abstract

**OBJECTIVE:**

To check the coverage of the HPV vaccine in women enrolled in health courses at a university in southwest Goiás, Brazil, and the factors associated with vaccination.

**METHODS:**

This is a cross-sectional study, including female university students of health courses, aged 18 years or more. A standardized and self-applying questionnaire was used. Participants who received two or more doses of the vaccine were considered immunized. Multiple analysis was performed using multinomial logistic regression.

**RESULT:**

We observed that, of the 1510 participants, 473 (31.3%) had two or more doses of HPV vaccine, 167 (11.0%) one dose and 870 (57.6%) were unvaccinated. Participants under 21 years of age and in socioeconomic stratum A were 2 times more likely to have received two or more doses of the vaccine (Prevalence Ratio = 1.95; 95%CI 1.40–2.70 and Prevalence Ratio = 2.09; 95%CI 1.39–3.13, respectively).

**CONCLUSIONS:**

The research revealed extensive possibility for interventions with the aim of achieving greater vaccination coverage among female university students. Even women with more knowledge and high economic stratum showed low vaccination coverage, suggesting that results of higher vaccine coverage can be obtained with vaccination carried out in a school environment.

## INTRODUCTION

Human papillomavirus (HPV) is considered the most common sexually transmitted infection (STI) worldwide, affecting more young adults and sexually active adolescents, probably due to behavioral factors, sociocultural attitudes and biological aspects^1^.

Sexual intercourse is the main form of HPV transmission, but one study showed that approximately 45% of adolescents contracted HPV before the onset of the first sexual intercourse with vaginal penetration^2^. It is likely that almost all sexually active women up to the age of 50 will have contact with at least one of the more than 130 HPV serotypes, but those under the age of 25, as female university students, have a higher chance of becoming infected, especially after sexarche^3^.

The prevalence of HPV in Brazilian women in 2010 in four regions (Southeast, South, Northeast and North) was between 13.7 and 54.3%^4^, however, partial data from a large multicenter Brazilian study showed a prevalence of 54.6% of HPV in the population, with 38.4% belonging to high-risk serotypes^5^.

Persistent HPV infections are associated with the appearance of almost all cervical cancers (CC) and high-grade cervical lesions, mainly by serotypes 16 and 18, involved in about 70% of these lesions^3,6^. In Brazil, in 2018 and 2019, about 16,370 new cases of cervical cancer were estimated, establishing a risk of 15.4 cases per 100,000 inhabitants and reaching the third place in the incidence of malignant tumors^7^.

Prophylactic vaccination against HPV is currently one of the main factors of prevention of CC^8^, its action reduces the number of infected people and spending on diagnosis and treatment. Vaccination is indicated mainly in adolescents who have not yet had the first sexual contact^3^, being recommended since 2006 in the USA and since 2007 in Australia.

In Brazil, HPV immunization started in 2014, initially for girls between 11 and 13 years, with the initial intention of reaching the goal of 80% of the female population. Even noting low coverage, in 2015 the *Programa Nacional de Imunizações* (PNI – National Immunization Program) expanded the vaccine for girls aged 9 to 13 years and for HIV patients aged 9 to 26 years. In 2016, vaccination began to be carried out in two doses (0 and 6 months), however, that same year, vaccine coverage was even lower. In 2017, we found that no Brazilian state reached 80% vaccine coverage against HPV. The highest coverages were observed in Roraima (67.6%), and in the Federal District (68.3%)^9^. In the state of Goiás, vaccination coverage for girls aged 9 to 14 years, during the years 2015 and 2018, was far below the goal established by the Ministry of Health, reaching only 13.6% for the first dose and 19.7% for the second dose^10^.

There are differences in adherence to HPV vaccination among children and adolescents, evidenced by lower coverage in children under 13 years of age, mainly because permission to vaccinate is associated with the willingness of the children’s parents. In order for parents to allow vaccination, they need to be informed about HPV infection and the benefits of the vaccine^11^.

When available almost exclusively in health facilities, HPV vaccination did not reach the goal of 80% coverage in most countries, according to a systematic review carried out in 120 countries. The exception was countries that adopted vaccination in the school environment as a strategy, such as Australia, Canada and the United Kingdom^12^.

Since there is an effective method of preventing the disease, but low vaccination coverage, it is important to check the factors associated with this picture and identify the population groups not yet immunized. Thus, this study found the coverage of vaccination against HPV and analyzed its associated factors, investigating a group of students enrolled in health courses at a university in the southwest of the state of Goiás.

## METHODS

This study was an excerpt of a research project designed to verify the living and health conditions of students enrolled in health courses at the Universidade do Rio Verde, GO (UniRV). In 2018, a cross-sectional study was conducted at UniRV campuses located in the southwest of Goiás in the municipalities of Rio Verde (176,424 inhabitants; HDI 0.754), Aparecida de Goiânia (455,657 inhabitants; HDI 0.718) and Goianésia (59,549 inhabitants; HDI 0.727), including students from nursing, dentistry, medicine, physiotherapy, pharmacy and physical education courses.

The project was presented and authorized by the course directors and disseminated via the *Sistema Educacional Integrado* (SEI – Integrated Education System), allowing access to regularly enrolled academics.

The questionnaire was applied to 2479 students, with 356 losses (14.3%). The study included all the female university students of the courses who were enrolled in the educational institution and aged 18 years old or older, a total of 1510 students.

The sample size of the research project allowed to estimate health problems with 50% prevalence (larger sample size required) with an accuracy of 2.2% and 95% confidence intervals. To detect associations, 10% were added for losses, to allow 80% of power to estimate a prevalence ratio of 1.13 with 95% confidence intervals. For this study, the sample was estimated with a confidence level of 95%, an error of three percentage points and a prevalence of 30%, requiring 795 participants.

A standardized, pre-coded and self-applying questionnaire was used. The questionnaire was prepared with an instruction manual to serve as a guide in case of questions in the filling or coding. The instrument was tested in another UniRV course. Professors distributed the questionnaires in the classroom, which after completion were deposited in an urn, without identifying the participants.

The outcomes were constructed from the question “Have you ever taken the HPV vaccine?”, recording as a response the number of doses. Thus, the variable was constructed with three categories: none; one dose; two or more doses. Participants who indicated they had received one or two or more doses of the HPV vaccine were analyzed. Demographic, socioeconomic variables, life habits, student characteristics and sexual behavior were also included.

The demographic variables were age (more than 23 years old; 21 to 23 years old; less than 21 years old); skin color (white; black; brown; other); marital status (with partner; without partner). The ABEP economic classification represented the socioeconomic variables and was categorized as strata: A, B, C, D, E. The economic ABEP classification is a combination of the possession of some material goods, the education of the head of the family, the presence of domestic employees and the availability of some public services at the place of residence^13^.

On life habits, variables were added as to physical activity (physically active or not); smoking habit (non-smokers; ex-smokers; smokers); consumption of illicit drugs in the last 30 days (no; yes); excessive use of alcohol (no; yes).

Participants who practiced at least 150 minutes of leisure time physical activity per week were considered physically active, based on the short version of the International Physical Activity Questionnaire (IPAQ)^14^. We considered as smoking habit the consumption of cigarettes and other forms, such as hookah, cigars, cigarillos, pipes, electronic cigarette, chewing tobacco and snuff. The consumption of illicit drugs included the use, in the last month, of marijuana, cocaine, crack, LSD, ecstasy, sniffin’ glue, *loló, lança-perfume*^a^. The excessive use of alcohol was considered by the application of the AUDIT scale, with 10 items and five-point Likert scale responses, when the scores reached 12 or more, were indicative of social problems related to alcohol^15^.

Student variables identified type of course (others; dentistry; medicine); failing on a subject (yes; no) and length of course (more than 5 years; 4 to 5 years; 2 to 4 years; up to 2 years).

Risky sexual behavior was measured by reference to sexually transmitted infection (STI) (no; yes); condom use in the first sexual intercourse (yes, no); number of partners in the last year (up to two; three or more); condom use in the last intercourse (yes; no)^16^. The variable “Have you had sexual relations with women?” (no; yes) was also analyzed.

The professors participating in the project were responsible for coding the questionnaires. Data was entered into the Epi-Data 3.1 program twice, for later comparison in order to eliminate the possibility of typos. The consistency and analysis of the data were carried out in the Stata *software*.

Data was analyzed according to the following steps. Initially, data were described by absolute and relative frequencies, of all independent variables, to portray the studied population.

For bivariate and multiple analyses, multinomial logistic regression was performed to investigate whether demographic, socioeconomic, lifestyle, student characteristics and sexual behavior variables were associated with one or two or more doses of HPV vaccine; the reference category was not having been vaccinated for HPV. The multiple final model was evaluated by the Brant test that did not violate the assumption of proportional odds^17^.

In the multiple analysis, variables entered the model hierarchically; this modeling considers possible conceptual bases valuing causal interrelationships. The variables that reached p < 0.20 in the bivariate analysis entered the model. The variables that reached the significance level of 5% remained in the model. In the hierarchical analysis model, the first level consisted of demographic and socioeconomic variables; the second level of life habits and student characteristics; and the third, of sexual behavior, all determining the outcome.

The research project was approved by the consubstantiated opinions of the research ethics committees, number 2,892,764, of the Universidade do Vale do Rio dos Sinos - UNISINOS, on September 13, 2018 and number 2,905,704, of the Universidade do Rio Verde, on September 19, 2018.

## RESULTS

Of the total of 1510 female participants, 473 (31.3%) received two or more doses of HPV vaccine, 167 (11.0%) received one dose, and another 870 (57.6%) were not vaccinated.

The distribution of the sample revealed the predominance of participants aged 21 to 23 years (48.6%), white (58.2%), without partner (88.6%) and inserted in the socioeconomic strata A and B (88.2%). Regarding life habits, the majority of the female university students were physically active (60.9%), non-smokers (90.1%), did not use illicit drugs 30 days prior to the survey (87.2%) and did not use alcohol excessively (67.1%). Student characteristics showed that most were in the Medicine course (69.7%), 15.4% had already failed a subject and 44.3% had more than 2 to 3 years of course time. Most of the interviewees had no history of STIs reported by a doctor (96.2%) and 83.5% of the participants revealed up to two sexual partners in the last year. Regarding the use of condoms, 79.9% of the female university students used condoms in their first sexual intercourse and 72.9% in the last. A significant majority did not have a relationship with other women (94.1%) (Table 1).

**Table 1:** Description of the sample and prevalence of HPV vaccination doses. Universidade de Rio Verde, 2019.

Variable	Total	No dose	1 dose	2 or more doses
			
	n (%)	n (%)	n (%)	n (%)
Age				
Over 23 years	392 (26.0)	257 (65.6)	35 (8.9)	100 (25.5)
From 21 to 23 years	734 (48.6)	437 (59.5)	76 (10.4)	221 (30.1)
Under 21 years	384 (25.4)	176 (45.8)	56 (14.6)	152 (39.6)
Skin color				
White	881 (58.2)	499 (56.6)	93 (10.6)	289 (32.8)
Black	47 (3.1)	28 (59.6)	6 (12.8)	13 (27.7)
Brown	521 (34.4)	312 (59.9)	59 (11.3)	150 (28.8)
Other	64 (4.2)	32 (50.0)	9 (14.1)	23 (35.9)
Marital status				
With partner	172 (11.4)	111 (64.5)	15 (8.7)	46 (26.7)
Without partner	1338 (88.6)	757 (56.6)	152 (11.4)	429 (32.1)
Economy class				
Classes C/D/E	172 (11.7)	105 (61.0)	26 (15.1)	41 (23.8)
Class B	693 (47.3)	441 (63.6)	76 (11.0)	176 (25.4)
Class A	599 (40.9)	291 (48.6)	60 (10.0)	248 (41.4)
Physical activity				
Not active	573 (39.1)	351 (61.3)	58 (10.1)	164 (28.6)
Active	892 (60.9)	492 (55.2)	101 (11.3)	299 (33.5)
Smoking habit				
Non-smoker	1336 (90.1)	773 (57.9)	146 (10.9)	417 (31.2)
Ex-smoker	80 (5.4)	43 (53.8)	9 (11.3)	28 (35.0)
Current smoker	66 (4.5)	39 (59.1)	7 (10.6)	20 (30.3)
Drug use in the last 30 days				
No	1264 (87.2)	740 (58.5)	141 (11.2)	383 (30.3)
Yes	186 (12.8)	98 (52.7)	22 (11.8)	66 (35.5)
Excessive alcohol use				
No	1013 (67.1)	595 (58.7)	110 (10.9)	308 (30.4)
Yes	497 (32.9)	274 (55.1)	56 (11.3)	167 (33.6)
Current course				
Others	135 (8.9)	91 (67.4)	22 (16.3)	22 (16.3)
Dentistry	323 (21.4)	182 (56.3)	51 (15.8)	90 (27.9)
Medicine	1052 (69.7)	596 (56.7)	94 (8.9)	362 (34.4)
Failure in a subject				
Yes	232 (15.4)	152 (65.5)	34 (14.7)	46 (19.8)
No	1278 (84.6)	717 (56.1)	133 (10.4)	428 (33.5)
Course time				
More than 5 years	65 (4.3)	47 (72.3)	4 (6.2)	14 (21.5)
More than 4 to 5 years	481 (31.9)	287 (59.7)	54 (11.2)	140 (29.1)
More than 2 to 4 years	667 (44.3)	397 (59.5)	62 (9.3)	208 (31.2)
Up to 2 years	294 (19.5)	136 (46.3)	47 (16.0)	111 (37.8)
STI referred by a doctor				
No	1443 (96.2)	831 (57.6)	160 (11.1)	452 (31.3)
Yes	57 (3.8)	32 (56.1)	6 (10.5)	19 (33.3)
Condom use in first intercourse				
Yes	1185 (79.9)	677 (57.1)	135 (11.4)	373 (31.5)
No	299 (20.1)	175 (58.5)	31 (10.4)	93 (31.1)
No. of partners in the last year				
Up to two	1244 (83.5)	723 (58.1)	133 (10.7)	388 (31.2)
Three or more	245 (16.5)	137 (55.9)	30 (12.2)	78 (31.8)
Condom use in the last intercourse				
Yes	1044 (72.9)	594 (56.9)	115 (11.0)	335 (32.1)
No	389 (27.1)	226 (58.1)	48 (12.3)	115 (29.6)
Have you ever had sexual relations with women?				
No	1320 (94.1)	773 (58.6)	141 (10.7)	406 (30.8)
Yes	83 (5.9)	42 (50.6)	12 (14.5)	29 (34.9)

STIs: sexually transmitted infections.

In the participants who received only one dose, the bivariate analysis showed association in the age group under 21 years and in those with up to two years of course time. In the multiple analysis of these same participants with one dose, an association was found only with those under 21 years of age (PR: 2.28; 95%CI: 1.43-3.63) (Table 2).

**Table 2:** Multinomial logistic regression of HPV vaccination (one dose) according to demographic, socioeconomic, student and lifestyle variables. Universidade de Rio Verde, 2019.

Variable	Bivariate analysis		Multiple analysis	
	
	PR (95%CI)	p	PR (95%CI)	p
Age		< 0.001		< 0.001^a^
Over 23 years	1		1	
From 21 to 23 years	1.27 (0.83–1.96)		1.23 (0.80–1.90)	
Under 21 years	2.33 (1.46–3.71)		2.28 (1.43–3.63)	
Skin color		0.606		
White	1			
Black	1.14 (0.46–2.85)			
Brown	1.01 (0.71–1.44)			
Other	1.50 (0.69–3.26)			
Marital status		0.171		0.256^a^
With partner	1		1	
Without partner	1.48 (0.84–2.61)		1.42 (0.80–2.51)	
Economy class		0.831		
Classes C/D/E	1			
Class B	0.69 (0.42–1.14)			
Class A	0.83 (0.49–1.38)			
Physical activity		0.225		
Not active	1			
Active	1.24 (0.87–1.76)			
Smoking habit		0.991		
Non-smoker	1			
Ex-smoker	1.10 (0.52–2.32)			
Current smoker	0.95 (0.41–2.16)			
Drug use in the last 30 days		0.517		
No	1			
Yes	1.17 (0.71–1.93)			
Excessive alcohol use		0.577		
No	1			
Yes	1.10 (0.77–1.57)			
Current course		0.009		0.014^b^
Others	1		1	
Dentistry	1.15 (0.66–2.02)		1.08 (0.61–1.91)	
Medicine	0.65 (0.39–1.09)		0.65 (0.38–1.11)	
Failure in a subject		0.377		
Yes	1			
No	0.82 (0.54–1.25)			
Course time		0.005		0.077^b^
More than 5 years	1		1	
More than 4 to 5 years	2.21 (0.76–6.39)		1.90 (0.65–5.54)	
More than 2 to 4 years	1.83 (0.63–5.27)		1.49 (0.50–4.41)	
Up to 2 years	4.06 (1.38–11.8)		3.01 (0.97–9.32)	
STI referred by the doctor		0.953		
No	1			
Yes	0.97 (0.40–2.36)			
Condom use in first intercourse		0.584		
Yes	1			
No	0.88 (0.58–1.35)			
No. of partners in the last year		0.434		
Up to two	1			
3 or more	1.19 (0.76–1.84)			
Condom use in the last intercourse		0.624		
Yes	1			
No	1.09 (0.75–1.58)			
Have you ever had sexual relations with women?		0.187		0.128^c^
No	1		1	
Yes	1.56 (0.80–3.04)		1.77 (0.90–3.48)	

STIs: sexually transmitted infections; PR: prevalence ratio; 95%CI: 95% confidence interval.

^a^ First level variables, adjusted to each other.

^b^ Second level variables, adjusted to each other and for age.

^c^ Third level variable, adjusted for age.

In turn, the participants who claimed to have received two or more doses were in the youngest age groups, classified into socioeconomic stratum A, physically active, from Dentistry and Medicine courses, without a history of failing a subject and with up to two years of course time. The variables marital status, drug use in the last 30 days and excessive alcohol use reached p-value < 0.20 and were led to multiple analysis (Table 3).

**Table 3:** Multinomial logistic regression of HPV vaccination (two or more doses) according to demographic, socioeconomic, student and lifestyle variables. Universidade de Rio Verde, 2019.

Variable	Bivariate analysis		Multiple analysis	
	
	PR (95%CI)	p	PR (95%CI)	p
Age		< 0.001		< 0.001ª
Over 23 years	1		1	
From 21 to 23 years	1.29 (0.98–1.72)		1.13 (0.84–1.51)	
Under 21 years	2.21 (1.61–3.04)		1.95 (1.40–2.70)	
Skin color		0.355		
White	1			
Black	0.80 (0.40–1.57)			
Brown	0.83 (0.65–1.05)			
Other	1.24 (0.71–2.16)			
Marital status		0.092		0.239ª
With partner	1		1	
Without partner	1.36 (0.95–1.96)		1.34 (0.91–1.96)	
Economy class		< 0.001		< 0.001ª
Classes C/D/E	1		1	
Class B	1.02 (0.68–1.52)		0.98 (0.65–1.48)	
Class A	2.18 (1.46–3.25)		2.09 (1.39–3.13)	
Physical activity		0.028		0.132^b^
Not active	1		1	
Active	1.30 (1.02–1.64)		1.25 (0.86–1.81)	
Smoking habit		0.859		
Non-smoker	1			
Ex-smoker	1.20 (0.73–1.97)			
Current smoker	0.95 (0.54–1.65)			
Drug use in the last 30 days		0.124		0.904^b^
No	1		1	
Yes	1.30 (0.93–1.82)		1.12 (0.65–1.95)	
Excessive alcohol use		0.176		0.351^b^
No	1		1	
Yes	1.17 (0.92–1.49)		1.01 (0.68–1.51)	
Current course		< 0.001		0.322^b^
Others	1		1	
Dentistry	2.04 (1.20–3.47)		1.63 (0.92–2.89)	
Medicine	2.51 (1.54–4.07)		1.61 (0.90–2.88)	
Failure in a subject		< 0.001		0.060^b^
Yes	1		1	
No	1.97 (1.38–2.80)		1.52 (0.90–2.36)	
Course time		< 0.001		0.296^b^
More than 5 years	1		1	
More than 4 to 5 years	1.63 (0.87–3.07)		1.44 (0.74–2.79)	
More than 2 to 4 years	1.75 (0.94–3.26)		1.23 (0.63–2.38)	
Up to 2 years	2.74 (1.43–5.23)		1.71 (0.84–3.49)	
STI referred by the doctor		0.767		
No	1			
Yes	1.09 (0.61–1.94)			
Condom use in first intercourse		0.802		
Yes	1			
No	0.96 (0.72–1.27)			
No. of partners in the last year		0.703		
Up to two	1			
3 or more	1.06 (0.78–1.43)			
Condom use in the last intercourse		0.441		
Yes	1			
No	0.90 (0.69–1.17)			
Have you ever had sex with women?		0.272		
No	1			
Yes	1.31 (0.80–2.14)			

STIs: sexually transmitted infections; PR: prevalence ratio; 95%CI: 95% confidence interval.

^a^ First level variables, adjusted to each other.

^b^ Second level variables, adjusted to each other and for age and economy class.

In the multiple analysis, the female university students who received two or more doses of vaccine were in the age group below 21 years (PR = 1.95; 95% CI: 1.40–2.70) inserted in socioeconomic stratum A (PR = 2.09; 95% CI: 1.39-3.13); the other variables lost statistical significance (Table 3).

## DISCUSSION

The vaccine coverage found in this study was lower than expected, however, higher than that observed in the state of Goiás in 2017^12^. Studies conducted in Europe and North America with university populations revealed low vaccine coverages^18^. In Canada, a study conducted at McGill University with 447 undergraduate students, with an average of 20 years, showed a prevalence of HPV vaccination of only 27.3% for one or more doses^18^. In 2013, another cross-sectional study conducted in Canada with 401 members of various courses at the University of Ottawa, through the internet, verified a prevalence of 35.9% of HPV vaccination for at least two doses^19^. In New York, in 2010, a study with 735 university students, including 381 women, showed prevalence of 56% for one dose and 44% for three doses of the vaccine^20^. A cross-sectional study conducted at the Midwestern University, conducted between 2007 and 2009, including 972 undergraduate students, found a prevalence of 49% for at least one vaccine dose^21^. In Marseille, France, a study involving 2018 high school and university students, aged between 15 and 45 years, showed a prevalence of 35.4% for the three doses^22^. The cross-sectional study with the highest coverage was carried out in Switzerland in 2017, with 409 women medical students, aged between 18 and 31 years, revealing a prevalence of 69.1%^23^.

In this study, higher coverage was expected, considering that the participants were students in the field of health, with greater access to information and knowledge about the severity of HPV infection and the importance of vaccination. However, one caveat must be considered regarding the knowledge acquired at the university and the age of the participants. HPV vaccination started in 2014, being initially offered free of charge only to girls aged 9 to 14 years^24^. Thus, unvaccinated female university students would need to be immunized in the private network. Even if 88.2% of the study participants were in socioeconomic strata A and B, the high costs of the vaccine in our country is a possible barrier to greater immunization. The results of the research point to the need to strengthen free vaccination against HPV in schools as an opportunity for prevention.

As most HPV vaccination campaigns around the world are usually targeted at children and adolescents up to 15 years, few studies have been found on vaccination coverage in college students over 18 years.

The hypothesis that a higher level of education added to greater purchasing power would favor a greater vaccination coverage was confirmed with this study, since the academic women belonging to socioeconomic stratum A were twice as vaccinated, when compared to those of strata C, D, E. Other studies have highlighted positive results in vaccination in relation to the socioeconomic situation. A cross-sectional study in Fujian, China, with 997 undergraduate students, showed that schooling (high knowledge scores) positively influenced vaccination, but the class or economic status did not exert significant influence on the intention to vaccinate^25^. The Childhood National Immunization Coverage Survey, conducted in 2013 in Canada, analyzed data from 5,213 women, relating the socioeconomic situation with vaccination, and concluded that the low levels of education and family income of parents were related to lower vaccine coverage, due to concerns about adverse effects and vaccine safety^26^. A cross-sectional study in the 50 American states with data from the 2014 and 2015 National Health Interview Survey, conducted with young people aged 18 to 26 years, showed that individuals without higher education, who did not have health insurance and with lower socioeconomic status were less likely to start and complete HPV vaccination^27^.

In our study, although the vaccination coverage of the socioeconomic stratum was far from the goal established by the Ministry of Health, it was almost twice as high as that found among the students of strata C, D and E, showing once again that the poorest women, who had less access to health services, were the least vaccinated. These data disghy8i9o70 agree with evidence pointing out that in Brazil, families with better socioeconomic conditions vaccinate their children less. This study showed that this may be different in the adolescent population and, especially, if the vaccine is not available for the age group studied^28^. This cycle can perpetuate the occurrence of cervical cancer among the poorest women, since they are the least vaccinated, who undergo screening tests of this pathology less regularly and, finally, have greater restriction of access to treatment^29^.

Another important fact is that the research revealed greater vaccine coverage among younger academics. A Canadian meta-analysis study showed that young people aged less than or equal to 18 years, were almost five times more likely to be vaccinated, compared to those aged over 18 years^30^. In Brazil, the routine vaccination has included girls aged 9 to 14 years, since 2014. Thus, due to the extent of knowledge about the importance of vaccination and perhaps because of the influence of government campaigns, we can expect increasing coverage from generation to generation, explaining this greater coverage in students under 21 years in this study.

Data also show that there is no influence of healthy lifestyle habits or risky sexual behaviors on the vaccination rate. However, due to the presence of professors at the time of filing the questionnaire (even if it was self-applied and without identification), it is necessary to consider that the participants may have omitted the occurrence of risky sexual behaviors.

The strengths of this study were the high number of participants, above the studies carried out with university students and the rigor in the conduct of field work and analysis.

The results reveal extensive possibilities for interventions in the population. The recognition of population subgroups with lower vaccination coverage can direct effective actions to achieve greater vaccination coverage among university students. Brazil has already used vaccination in schools at other times with excellent results, with vaccination campaigns against mumps, rubella and measles, with coverage of 95%^28^. Perhaps, the provision of vaccines in schools, in addition to health units, would shift the vaccine from the context of disease to the environment of prevention and knowledge that the school can develop better, explaining and integrating students and parents, with the support of teachers engaged in vaccination and trained to give information.

## Footnotes

a Translator’s Note: In Brazil, *loló* and *lança-perfume* are slangs or popular names for clandestinely prepared narcotics based on chloroform and ether.
