# Supplementary material for: Vaccination coverage against human papillomavirus (HPV) and associated factors in female academics from a university in southwestern Goiás, Brazil
Source: Rev Saude Publica. 2021 Oct 13;55:65. doi: 10.11606/s1518-8787.2021055003144 (PMC8522710; doi:10.11606/s1518-8787.2021055003144)
Supplement: Translation

# Translation: Cobertura vacinal contra o papiloma vírus humano (HPV) e fatores associados em acadêmicas de uma universidade do sudoeste goiano

**Authors:** Paulo Sérgio de Oliveira, Carla Vitola Gonçalves, Guilherme Watte, Juvenal Soares Dias da Costa

**Affiliations:** I Brasil Universidade de Rio Verde. Faculdade de Medicina. Rio Verde, GO, Brasil; II Brasil Universidade Federal do Rio Grande. Faculdade de Medicina. Rio Grande, RS, Brasil; III Brasil Universidade Católica do Rio Grande do Sul. Programa de Pós-Graduação em Medicina e Ciências da Saúde da Pontifícia, Porto Alegre, RS, Brasil; IV Brasil Universidade do Vale do Rio dos Sinos. Programa de Pós-Graduação em Saúde Coletiva. São Leopoldo, RS, Brasil

**Keywords:** Adulto Jovem, Estudantes de Ciências da Saúde, Infecções por Papillomavirus, prevenção & controle, Vacinas contra Papillomavirus, provisão & distribuição, Cobertura Vacinal

## Abstract

**OBJETIVO:**

Verificar a cobertura da vacina contra o HPV em mulheres matriculadas em cursos da área de saúde de uma universidade do sudoeste do Estado de Goiás e os fatores associados à vacinação.

**MÉTODOS:**

Trata-se de estudo transversal, incluindo universitárias dos cursos da área de saúde, com 18 anos ou mais. Foi utilizado questionário padronizado e autoaplicável. As participantes que receberam duas ou mais doses da vacina foram consideradas como imunizadas. A análise múltipla foi realizada por meio de regressão logística multinomial.

**RESULTADOS:**

Observou-se que, das 1510 participantes, 473 (31,3%) com duas ou mais doses de vacina contra o HPV, 167 (11,0%) com uma dose e 870 (57,6%) não vacinadas. As participantes com menos de 21 anos e inseridas no estrato socioeconômico A tinham 2 vezes mais chance de terem recebido duas doses ou mais da vacina (Razão de Prevalência = 1,95; IC95% 1,40–2,70 e Razão de Prevalência = 2,09; IC95% 1.39–3,13, respectivamente).

**CONCLUSÕES:**

A pesquisa revelou extensa possibilidade para intervenções com o objetivo de atingir maior cobertura vacinal entre as universitárias. Mesmo mulheres com mais conhecimento e de estrato econômico elevado apresentaram baixa cobertura vacinal, sugerindo que resultados de cobertura vacinal maior podem ser obtidos com a vacinação realizada em ambiente escolar.

## INTRODUÇÃO

O Papiloma vírus Humano (HPV) é considerado a infecção sexualmente transmissível (IST) mais frequente em todo o mundo, acometendo mais adultos jovens e adolescentes sexualmente ativos, provavelmente devido a fatores comportamentais, atitudes socioculturais e aspectos biológicos^1^.

Relações sexuais são a principal forma de transmissão do HPV, porém um estudo mostrou que aproximadamente 45% das adolescentes contraíram o HPV antes do início da primeira relação sexual com penetração vaginal^2^. Provavelmente, quase a totalidade das mulheres sexualmente ativas até os 50 anos terão contato com pelo menos um dos mais de 130 sorotipos de HPV, mas aquelas com idade inferior a 25 anos, como estudantes universitárias, têm maiores possibilidades de se infectar, principalmente após a sexarca^3^.

As prevalências de HPV em mulheres brasileiras, em 2010, em quatro regiões (Sudeste, Sul, Nordeste e Norte) ficaram entre 13,7 e 54,3%^4^, contudo, dados parciais de um grande estudo brasileiro multicêntrico, mostrou prevalência de 54,6% de HPV na população, sendo 38,4% pertencendo aos sorotipos de alto risco^5^.

Infecções persistentes por HPV estão associadas ao aparecimento de quase todos os cânceres do colo uterino (CCU) e de lesões cervicais de alto grau, principalmente pelos sorotipos 16 e 18, envolvidos em cerca de 70% dessas lesões^3,6^. No Brasil, em 2018 e 2019, foram estimados cerca de 16.370 novos casos de câncer cervical, estabelecendo um risco de 15,4 casos a cada 100.000 habitantes e atingindo o terceiro lugar na incidência dos tumores malignos^7^.

A vacinação profilática contra o HPV é atualmente um dos principais fatores de prevenção do CCU^8^, sua ação reduz o número de pessoas infectadas e os gastos com diagnóstico e tratamentos. A vacinação está indicada principalmente em adolescentes que ainda não tiveram o primeiro contato sexual^3^, sendo recomendada desde 2006 nos EUA e, a partir de 2007, na Austrália.

No Brasil, a imunização contra o HPV foi iniciada em 2014, inicialmente para meninas entre 11 e 13 anos, com intenção inicial de atingir a meta de 80% da população feminina. Mesmo constatando baixas coberturas, em 2015 o Programa Nacional de Imunizações (PNI) ampliou a vacina para meninas de 9 a 13 anos e para portadoras de HIV de 9 a 26 anos. Em 2016, a vacinação passou a ser realizada em duas doses (0 e 6 meses), porém, nesse mesmo ano, as coberturas vacinais foram ainda mais baixas. Em 2017, verificou-se que nenhum estado brasileiro atingiu a cobertura vacinal de 80% contra o HPV. As maiores coberturas foram observadas em Roraima (67,6%), e no Distrito Federal (68,3%)^9^. No Estado de Goiás, a cobertura vacinal para as meninas com idade entre 9 e 14 anos, durante os anos de 2015 e 2018, ficou muito abaixo da meta estabelecida pelo Ministério da Saúde, atingindo somente 13,6% para a primeira dose e 19,7% para a segunda dose^10^.

Há diferenças na adesão à vacinação contra HPV entre crianças e adolescentes, evidenciadas com uma menor cobertura em menores de 13 anos, principalmente porque a permissão à vacinação está associada à disposição dos pais das crianças. Para que os pais permitam a vacinação, é necessário que sejam informados sobre a infecção por HPV e os benefícios da vacina^11^.

Quando disponibilizada quase exclusivamente em estabelecimentos de saúde, a vacinação contra o HPV não atingiu o objetivo de 80% de cobertura na maior parte dos países, segundo revisão sistemática realizada em 120 países. A exceção foi verificada em países que adotaram como estratégia a vacinação no ambiente escolar, como Austrália, Canadá e no Reino Unido^12^.

Uma vez que há um método eficaz de prevenção da doença, mas baixa cobertura vacinal, é importante verificar os fatores associados a esse quadro e identificar os grupos populacionais ainda não imunizados. Assim, este estudo constatou a cobertura de vacinação contra o HPV e analisou seus fatores associados, investigando um grupo de estudantes matriculadas em cursos da área de saúde de uma universidade do sudoeste do Estado de Goiás.

## MÉTODOS

Esse estudo foi um recorte de um Projeto de Pesquisa elaborado para verificar as condições de vida e saúde de estudantes matriculados em cursos da área de saúde da Universidade de Rio Verde, GO (UniRV). Em 2018, foi realizado estudo transversal nos campi da UniRV localizados no sudoeste de Goiás nos municípios de Rio Verde (176.424 habitantes; IDH 0,754), Aparecida de Goiânia (455.657 habitantes; IDH 0,718) e Goianésia (59,549 habitantes; IDH 0,727), incluindo alunos dos cursos de Enfermagem, Odontologia, Medicina, Fisioterapia, Farmácia e Educação Física.

O Projeto foi apresentado e autorizado pelos diretores dos cursos e divulgados via Sistema Educacional Integrado (SEI), permitindo acesso aos acadêmicos regularmente matriculados.

O questionário foi aplicado em 2479 estudantes, tendo 356 perdas (14,3%). No estudo foram incluídas todas as universitárias dos cursos que estavam matriculadas na instituição de ensino e que tivessem 18 anos ou mais, num total de 1510 alunas.

O tamanho da amostra do projeto de pesquisa permitiu estimar agravos de saúde com 50% de prevalência (maior tamanho de amostra necessário) com uma precisão de 2,2% e intervalos de confiança de 95%. Para detectar associações foram adicionados 10% para perdas, para permitir 80% de poder para estimar razão de prevalência de 1,13 com intervalos de confiança de 95%. Para o presente estudo a amostra foi estimada com nível de confiança de 95%, erro de três pontos percentuais e prevalência de 30% sendo necessárias 795 participantes.

Foi utilizado questionário padronizado, pré-codificado e autoaplicável. O questionário foi elaborado com manual de instruções para servir de guia no caso de dúvidas no preenchimento ou codificação. O instrumento foi testado em outro curso da UniRV. Os professores distribuíam os questionários em sala de aula, que após preenchimento eram depositados em urna, não permitindo identificação dos participantes.

Os desfechos foram construídos a partir da pergunta “você já tomou vacina contra HPV?”, registrando-se como resposta o número de doses. Dessa forma, a variável foi construída com três categorias: nenhuma; uma dose; duas ou mais doses. As participantes que indicaram ter recebido uma ou duas ou mais doses da vacina contra HPV foram analisadas. Também se incluiu variáveis demográficas, socioeconômicas, hábitos de vida, características discentes e de comportamento sexual.

As variáveis demográficas foram idade (maior de 23 anos; de 21 a 23 anos; menor de 21 anos); cor da pele referida (branca; preta; parda; outra); situação conjugal (com companheiro; sem companheiro). A classificação econômica da ABEP representou as variáveis socioeconômicas e foi categorizada como estratos: A, B, C, D, E. A classificação econômica da ABEP é uma combinação entre a posse de alguns bens materiais, a escolaridade do chefe da família, a presença de empregados domésticos e a disponibilidade de alguns serviços públicos no local de residência^13^.

Sobre os hábitos de vida, acrescentaram-se as variáveis quanto a atividade física (fisicamente ativos ou não); hábito de fumar (não fumantes; ex-fumantes; fumantes); consumo de drogas ilícitas nos últimos 30 dias (não; sim); uso excessivo de álcool (não; sim).

Considerou-se ativas fisicamente as participantes que praticavam no mínimo 150 minutos semanais de atividade física no lazer, com base na versão curta do Questionário Internacional de Atividade Física (IPAQ)^14^. Como hábito de fumar foi considerado o consumo de cigarro e outras formas, como narguilé, charutos, cigarrilhas, cachimbos, cigarro eletrônico, fumo de mascar e rapé. O consumo de drogas ilícitas incluiu o uso, no último mês, de maconha, cocaína, crack, LSD, ecstasy, cola, loló, lança perfumes. O uso excessivo de álcool foi considerado pela aplicação da escala AUDIT, com 10 itens e respostas em escala likert de cinco pontos, quando os escores atingiram 12 ou mais, foram indicativos de problemas sociais relacionados ao álcool^15^.

As variáveis discentes identificaram tipo de curso (outros; odontologia; medicina); ocorrência de reprovação (sim; não) e tempo de curso (mais de 5 anos; mais de 4 a 5 anos; mais de 2 a 4 anos; até 2 anos).

Para medir o comportamento sexual de risco foi considerado pela referência a infecção sexualmente transmissível (IST) (não; sim); pelo uso de preservativo na primeira relação sexual (sim, não); número de parceiros no último ano (até dois; três ou mais); uso de preservativo na última relação (sim; não)^16^. Foi analisada ainda a variável “se já manteve relações sexuais com mulheres” (não; sim).

Os professores participantes do Projeto se responsabilizaram pela codificação dos questionários. A entrada dos dados no programa Epi-Data 3.1 foi realizada duas vezes, para posterior comparação de modo a eliminar a possibilidade de erros de digitação. A consistência e a análise dos dados foram realizadas no *software* Stata.

A análise dos dados seguiu os seguintes passos. Inicialmente, os dados foram descritos por meio das frequências absolutas e relativas, de todas as variáveis independentes, para retratar a população estudada.

Para as análises bivariada e múltipla foi realizada regressão logística multinomial para investigar se as variáveis demográficas, socioeconômicas, hábitos de vida, características discentes e de comportamento sexual estavam associadas à realização de uma ou duas ou mais doses de vacina para o HPV, sendo a categoria de referência não ter feito vacina para o HPV. O modelo final múltiplo foi avaliado por meio do teste de Brant que não violou o pressuposto de chances proporcionais^17^.

Na análise múltipla, as variáveis ingressaram no modelo de forma hierarquizada, essa modelagem leva em consideração possíveis bases conceituais valorizando as interrelações causais. Ingressaram no modelo as variáveis que atingiram valor de p < 0,20 na análise bivariada. Permaneceram no modelo as variáveis que atingiram o nível de significância de 5%. No modelo hierarquizado de análise o primeiro nível estava constituído pelas variáveis demográficas e socioeconômicas, no segundo nível estavam os hábitos de vida e as características discentes e, no terceiro nível, as de comportamento sexual, todas determinando o desfecho.

O projeto de pesquisa foi aprovado mediante os pareceres consubstanciados dos comitês de ética em pesquisa, número 2.892.764, da Universidade do Vale do Rio dos Sinos- UNISINOS, em 13 de setembro de 2018 e número 2.905.704, da Universidade de Rio Verde, em 19 de setembro de 2018.

## RESULTADOS

Do total de 1510 participantes do sexo feminino, 473 (31,3%) receberam duas ou mais doses de vacina contra o HPV, 167 (11,0%) receberam uma dose e outras 870 (57,6%) não foram vacinadas.

A distribuição da amostra revelou o predomínio das participantes de 21 a 23 anos (48,6%), de pele branca (58,2%), sem companheiro (88,6%) e inseridas nos estratos socioeconômicos A e B (88,2%). Em relação aos hábitos de vida, a maioria das universitárias era ativa fisicamente (60,9%), não fumante (90,1%), não usou de drogas ilícitas 30 dias anteriores à pesquisa (87,2%) e não fazia uso excessivo de álcool (67,1%). As características discentes mostraram que a maioria estava no curso de Medicina (69,7%), 15,4% já tinha sido reprovada e 44,3% apresentavam mais de 2 a 3 anos de tempo de curso. A maior parte das entrevistadas não apresentou passado de IST referida por algum médico (96,2%) e 83,5% das participantes revelaram até dois parceiros sexuais no último ano. Sobre o uso de preservativos, 79,9% das universitárias usaram preservativo na sua primeira relação sexual e 72,9% na última. Um número expressivo não manifestou relacionamento com outras mulheres (94,1%) (Tabela 1).

**Tabela 1:** Descrição da amostra e prevalências das doses de vacinação contra HPV. Universidade de Rio Verde, 2019.

Variável	Total	Nenhuma dose	1 dose	2 ou mais doses
			
	n (%)	n (%)	n (%)	n (%)
Idade				
Maior de 23 anos	392 (26,0)	257 (65,6)	35 (8,9)	100 (25,5)
De 21 a 23 anos	734 (48,6)	437 (59,5)	76 (10,4)	221 (30,1)
Menor de 21 anos	384 (25,4)	176 (45,8)	56 (14,6)	152 (39,6)
Cor da pele				
Branca	881 (58,2)	499 (56,6)	93 (10,6)	289 (32,8)
Preta	47 (3,1)	28 (59,6)	6 (12,8)	13 (27,7)
Parda	521 (34,4)	312 (59,9)	59 (11,3)	150 (28,8)
Outra	64 (4,2)	32 (50,0)	9 (14,1)	23 (35,9)
Situação conjugal				
Com companheiro	172 (11,4)	111 (64,5)	15 (8,7)	46 (26,7)
Sem companheiro	1338 (88,6)	757 (56,6)	152 (11,4)	429 (32,1)
Classe econômica				
Classes C/D/E	172 (11,7)	105 (61,0)	26 (15,1)	41 (23,8)
Classe B	693 (47,3)	441 (63,6)	76 (11,0)	176 (25,4)
Classe A	599 (40,9)	291 (48,6)	60 (10,0)	248 (41,4)
Atividade física				
Não ativos	573 (39,1)	351 (61,3)	58 (10,1)	164 (28,6)
Ativos	892 (60,9)	492 (55,2)	101 (11,3)	299 (33,5)
Hábito de fumar				
Não fumante	1336 (90,1)	773 (57,9)	146 (10,9)	417 (31,2)
Ex-fumante	80 (5,4)	43 (53,8)	9 (11,3)	28 (35,0)
Fumante atual	66 (4,5)	39 (59,1)	7 (10,6)	20 (30,3)
Uso de drogas nos últimos 30 dias				
Não	1264 (87,2)	740 (58,5)	141 (11,2)	383 (30,3)
Sim	186 (12,8)	98 (52,7)	22 (11,8)	66 (35,5)
Uso excessivo de álcool				
Não	1013 (67,1)	595 (58,7)	110 (10,9)	308 (30,4)
Sim	497 (32,9)	274 (55,1)	56 (11,3)	167 (33,6)
Curso atual				
Outros	135 (8,9)	91 (67,4)	22 (16,3)	22 (16,3)
Odontologia	323 (21,4)	182 (56,3)	51 (15,8)	90 (27,9)
Medicina	1052 (69,7)	596 (56,7)	94 (8,9)	362 (34,4)
Reprovação				
Sim	232 (15,4)	152 (65,5)	34 (14,7)	46 (19,8)
Não	1278 (84,6)	717 (56,1)	133 (10,4)	428 (33,5)
Tempo de curso				
Mais de 5 anos	65 (4,3)	47 (72,3)	4 (6,2)	14 (21,5)
Mais de 4 a 5 anos	481 (31,9)	287 (59,7)	54 (11,2)	140 (29,1)
Mais de 2 a 4 anos	667 (44,3)	397 (59,5)	62 (9,3)	208 (31,2)
Até 2 anos	294 (19,5)	136 (46,3)	47 (16,0)	111 (37,8)
IST referida pelo médico				
Não	1443 (96,2)	831 (57,6)	160 (11,1)	452 (31,3)
Sim	57 (3,8)	32 (56,1)	6 (10,5)	19 (33,3)
Uso de preservativo na primeira relação				
Sim	1185 (79,9)	677 (57,1)	135 (11,4)	373 (31,5)
Não	299 (20,1)	175 (58,5)	31 (10,4)	93 (31,1)
Nº parceiros no último ano				
Até dois	1244 (83,5)	723 (58,1)	133 (10,7)	388 (31,2)
Três ou mais	245 (16,5)	137 (55,9)	30 (12,2)	78 (31,8)
Uso de preservativo na última relação				
Sim	1044 (72,9)	594 (56,9)	115 (11,0)	335 (32,1)
Não	389 (27,1)	226 (58,1)	48 (12,3)	115 (29,6)
Já teve relações com mulheres?				
Não	1320 (94,1)	773 (58,6)	141 (10,7)	406 (30,8)
Sim	83 (5,9)	42 (50,6)	12 (14,5)	29 (34,9)

IST: infecções sexualmente transmissíveis.

Nas participantes que receberam apenas uma dose a análise bivariada mostrou associação na faixa etária menor de 21 anos e naquelas com até dois anos de tempo de curso. Na análise múltipla dessas mesmas participantes com uma dose, constatou-se associação apenas com aquelas menores de 21 anos (RP: 2,28; IC95%: 1,43–3,63) (Tabela 2).

**Tabela 2:** Regressão logística multinomial de vacinação contra HPV (uma dose) de acordo com variáveis demográficas, socioeconômicas, discentes e hábitos de vida. Universidade de Rio Verde, 2019.

Variáveis	Análise bivariada		Análise múltipla	
	
	RP (IC95%)	p	RP (IC95%)	p
Idade		< 0,001		< 0,001^a^
Maior de 23 anos	1		1	
De 21 a 23 anos	1,27 (0,83–1,96)		1,23 (0,80–1,90)	
Menor de 21 anos	2,33 (1,46–3,71)		2,28 (1,43–3,63)	
Cor da pele		0,606		
Branca	1			
Preta	1,14 (0,46–2,85)			
Parda	1,01 (0,71–1,44)			
Outra	1,50 (0,69–3,26)			
Situação conjugal		0,171		0,256^a^
Com companheiro	1		1	
Sem companheiro	1,48 (0,84–2,61)		1,42 (0,80–2,51)	
Classe econômica		0,831		
Classes C/D/E	1			
Classe B	0,69 (0,42–1,14)			
Classe A	0,83 (0,49–1,38)			
Atividade física		0,225		
Não ativos	1			
Ativos	1,24 (0,87–1,76)			
Hábito de fumar		0,991		
Não fumante	1			
Ex-fumante	1,10 (0,52–2,32)			
Fumante atual	0,95 (0,41–2,16)			
Uso de drogas nos últimos 30 dias		0,517		
Não	1			
Sim	1,17 (0,71–1,93)			
Uso excessivo de álcool		0,577		
Não	1			
Sim	1,10 (0,77–1,57)			
Curso atual		0,009		0,014^b^
Outros	1		1	
Odontologia	1,15 (0,66–2,02)		1,08 (0,61–1,91)	
Medicina	0,65 (0,39–1,09)		0,65 (0,38–1,11)	
Reprovação		0,377		
Sim	1			
Não	0,82 (0,54–1,25)			
Tempo de curso		0,005		0,077^b^
Mais de 5 anos	1		1	
Mais de 4 a 5 anos	2,21 (0,76–6,39)		1,90 (0,65–5,54)	
Mais de 2 a 4 anos	1,83 (0,63–5,27)		1,49 (0,50–4,41)	
Até 2 anos	4,06 (1,38–11,8)		3,01 (0,97–9,32)	
IST referida pelo médico		0,953		
Não	1			
Sim	0,97 (0,40–2,36)			
Uso de preservativo na primeira relação		0,584		
Sim	1			
Não	0,88 (0,58–1,35)			
N° de parceiros no último ano		0,434		
Até dois	1			
3 ou mais	1,19 (0,76–1,84)			
Uso de preservativo na última relação		0,624		
Sim	1			
Não	1,09 (0,75–1,58)			
Já teve relações com mulheres?		0,187		0,128^c^
Não	1		1	
Sim	1,56 (0,80–3,04)		1,77 (0,90–3,48)	

IST: infecções sexualmente transmissíveis; RP: razão de prevalência; IC95%: intervalo de confiança de 95%.

^a^ Variáveis do primeiro nível, ajustadas entre si.

^b^ Variáveis do segundo nível, ajustadas entre si e para idade.

^c^ Variável do terceiro nível, ajustada para idade.

Por sua vez, participantes que afirmaram ter recebido duas ou mais doses estavam nas faixas etárias mais jovens, naquelas classificadas no estrato socioeconômico A, nas ativas fisicamente, nas alunas dos cursos de odontologia e medicina, sem histórico de reprovação e com até dois anos de tempo de curso. As variáveis situação conjugal, uso de drogas nos últimos 30 dias e uso excessivo de álcool alcançaram p-valor < 0,20 e foram conduzidas à análise múltipla (Tabela 3).

**Tabela 3:** Regressão logística multinomial de vacinação contra HPV (duas ou mais doses) de acordo com variáveis demográficas, socioeconômicas, discentes e hábitos de vida. Universidade de Rio Verde, 2019.

Variáveis	Análise bivariada		Análise múltipla	
	
	RP (IC95%)	p	RP (IC95%)	p
Idade		< 0,001		< 0,001ª
Maior de 23 anos	1		1	
De 21 a 23 anos	1,29 (0,98–1,72)		1,13 (0,84–1,51)	
Menor de 21 anos	2,21 (1,61–3,04)		1,95 (1,40–2,70)	
Cor da pele		0,355		
Branca	1			
Preta	0,80 (0,40–1,57)			
Parda	0,83 (0,65–1,05)			
Outra	1,24 (0,71–2,16)			
Situação conjugal		0,092		0,239ª
Com companheiro	1		1	
Sem companheiro	1,36 (0,95–1,96)		1,34 (0,91–1,96)	
Classe econômica		< 0,001		< 0,001ª
Classes C/D/E	1		1	
Classe B	1,02 (0,68–1,52)		0,98 (0,65–1,48)	
Classe A	2,18 (1,46–3,25)		2,09 (1,39–3,13)	
Atividade física		0,028		0,132^b^
Não ativos	1		1	
Ativos	1,30 (1,02–1,64)		1,25 (0,86–1,81)	
Hábito de fumar		0,859		
Não fumante	1			
Ex–fumante	1,20 (0,73–1,97)			
Fumante atual	0,95 (0,54–1,65)			
Uso de drogas nos últimos 30 dias		0,124		0,904^b^
Não	1		1	
Sim	1,30 (0,93–1,82)		1,12 (0,65–1,95)	
Uso excessivo de álcool		0,176		0,351^b^
Não	1		1	
Sim	1,17 (0,92–1,49)		1,01 (0,68–1,51)	
Curso atual		< 0,001		0,322^b^
Outros	1		1	
Odontologia	2,04 (1,20–3,47)		1,63 (0,92–2,89)	
Medicina	2,51 (1,54–4,07)		1,61 (0,90–2,88)	
Reprovação		< 0,001		0,060^b^
Sim	1		1	
Não	1,97 (1,38–2,80)		1,52 (0,90–2,36)	
Tempo de curso		< 0,001		0,296^b^
Mais de 5 anos	1		1	
Mais de 4 a 5 anos	1,63 (0,87–3,07)		1,44 (0,74–2,79)	
Mais de 2 a 4 anos	1,75 (0,94–3,26)		1,23 (0,63–2,38)	
Até 2 anos	2,74 (1,43–5,23)		1,71 (0,84–3,49)	
IST referida pelo médico		0,767		
Não	1			
Sim	1,09 (0,61–1,94)			
Uso de preservativo na primeira relação		0,802		
Sim	1			
Não	0,96 (0,72–1,27)			
N° de parceiros no último ano		0,703		
Até dois	1			
3 ou mais	1,06 (0,78–1,43)			
Uso de preservativo na última relação		0,441		
Sim	1			
Não	0,90 (0,69–1,17)			
Já teve relações com mulheres?		0,272		
Não	1			
Sim	1,31 (0,80–2,14)			

^a^ Variáveis do primeiro nível, ajustadas entre si.

^b^ Variáveis do segundo nível, ajustadas entre si e para idade e classe econômica.

IST – infecções sexualmente transmissíveis.

RP – razão de prevalência IC 95% - Intervalo de confiança à 95%.

Na análise múltipla, as universitárias que receberam duas ou mais doses de vacina estavam na faixa etária menor de 21 anos (RP = 1,95; IC95%: 1,40–2,70) e nas inseridas no estrato socioeconômico A (RP = 2,09; IC95%: 1,39–3,13), as demais variáveis perderam significância estatística (Tabela 3).

## DISCUSSÃO

A cobertura vacinal encontrada no presente estudo foi inferior à esperada, contudo, superior à observada no Estado de Goiás em 2017^12^. Estudos conduzidos na Europa e na América do Norte com populações universitárias revelaram baixas coberturas da vacina^18^. No Canadá, estudo realizado na Universidade McGill com 447 estudantes de graduação, com média de 20 anos, evidenciou prevalência de vacinação contra o HPV de apenas 27,3% para uma ou mais doses^18^. Em 2013, outro estudo transversal realizado no Canadá com 401 integrantes de vários cursos da Universidade de Ottawa, por meio da internet, verificou prevalência de 35,9% de vacinação contra o HPV para pelo menos duas doses^19^. Em Nova Iorque, em 2010, estudo com 735 universitários, incluindo 381 mulheres, mostrou prevalência de 56% para uma dose e 44% para três doses da vacina^20^. Pesquisa realizada na Universidade do Meio Oeste, também de delineamento transversal, realizado entre 2007 e 2009, incluindo 972 alunas de graduação, encontrou prevalência de 49% de pelo menos uma dose vacinal^21^. Em Marselha, na França, estudo envolvendo 2018 estudantes do ensino médio e universitários, nas idades entre 15 e 45 anos, apresentou prevalência de 35,4% para as três doses^22^. O estudo transversal com cobertura mais elevada foi realizado na Suíça em 2017, com 409 mulheres estudantes de medicina, com idades entre 18 e 31 anos, revelando prevalência de 69,1%^23^.

Neste estudo, esperava-se cobertura mais elevada, levando em consideração que as participantes eram estudantes das áreas de saúde, com maior acesso a informação e conhecimento sobre a gravidade da infecção por HPV e a importância da vacinação. Contudo, é preciso fazer uma ressalva em consideração aos conhecimentos adquiridos na universidade e a idade das participantes. A vacinação contra o HPV teve início em 2014, sendo ofertada inicialmente e gratuitamente somente para meninas de 9 a 14 anos^24^. Assim, as universitárias não vacinadas, precisariam ser imunizadas na rede privada. Mesmo que 88,2% das participantes do estudo estivessem nos estratos socioeconômicos A e B, os altos custos da vacina em nosso país é uma possível barreira para uma imunização maior. Os resultados da pesquisa apontam para a necessidade de se reforçar a vacinação gratuita contra o HPV nas escolas como oportunidade de prevenção.

Como a maioria das campanhas vacinação contra o HPV em todo o mundo costuma ser direcionada a crianças e adolescentes até 15 anos, foram encontrados poucos estudos sobre a cobertura de vacinação em universitários acima de 18 anos.

A hipótese de que um maior nível de escolaridade somado a maior poder aquisitivo favoreceria uma maior cobertura vacinal foi confirmada com este estudo, pois as acadêmicas pertencentes ao estrato socioeconômico A, foram duas vezes mais vacinadas, quando comparadas às dos estratos C, D, E. Outros estudos destacaram resultados positivos na vacinação em relação à situação socioeconômica. Estudo transversal em Fujian, na China, com 997 estudantes de graduação, evidenciou que a escolaridade (altos escores de conhecimento) influenciou positivamente na vacinação, porém a classe ou *status* econômico, não exerceu influência significativa sobre a intenção de vacinar-se^25^. A Pesquisa Nacional de Cobertura e Imunizações Infantis, realizada em 2013 no Canadá, analisou dados de 5.213 mulheres, relacionando a situação socioeconômica com o recebimento vacinal, concluiu que os baixos níveis de educação e renda familiar dos pais estavam relacionados a menores coberturas vacinais, devido a preocupações com efeitos adversos e segurança da vacina^26^. Estudo transversal em 50 estados americanos com dados da Pesquisa Nacional de Entrevistas em Saúde, de 2014 e 2015, realizado com jovens de 18 a 26 anos, mostrou que indivíduos sem ensino superior, que não possuíam seguro de saúde e com situação socioeconômica mais baixa tinham menor probabilidade de iniciar e concluir a vacinação contra o HPV^27^.

No presente estudo, apesar da cobertura vacinal do estrato socioeconômico A estar longe da meta estabelecida pelo Ministério da Saúde, ela foi quase duas vezes maior do que a encontrada entre as estudantes do estrato C, D, E, mostrando mais uma vez que as mulheres mais pobres, que possuíam menor acesso aos serviços de saúde foram as menos vacinadas. Esses dados vão de encontro com evidências apontando que no Brasil, as famílias com melhores condições socioeconômicas vacinam menos suas crianças. O presente estudo mostrou que pode ser diferente na população adolescente e, principalmente, se a vacina não estiver disponível para o grupo etário estudado^28^. Esse ciclo pode perpetuar a ocorrência de câncer de colo uterino entre as mulheres mais pobres, já que são as menos vacinadas, que se submetem com menor regularidade aos exames de rastreio dessa patologia e, por fim, apresentam maior restrição de acesso ao tratamento^29^.

Outro dado importante é que a pesquisa revelou maior cobertura vacinal entre as acadêmicas mais jovens. Estudo canadense de meta-análise mostrou que jovens em idades menores ou iguais a 18 anos, tiveram quase cinco vezes mais chance de serem vacinados, em comparação aos de idade acima de 18 anos^30^. No Brasil, a rotina de vacinação tem incluído meninas de 9 a 14 anos, desde 2014. Assim, devido a extensão do conhecimento sobre a importância da vacinação e talvez por influência das campanhas governamentais, pode-se esperar coberturas crescentes de geração em geração, explicando-se essa maior cobertura em alunas menores de 21 anos no presente estudo.

Os dados mostram ainda que não há influência de hábitos de vida saudáveis ou comportamentos sexuais de risco sobre o índice de vacinação. Porém, devido a presença de professores no momento do depósito do questionário (mesmo que fosse autoaplicado e sem identificação), é preciso considerar que as participantes possam ter omitido a ocorrência de comportamentos sexuais de risco.

Os pontos fortes desse estudo foram o elevado número de participantes, acima dos estudos realizados com universitários e o rigor na condução do trabalho de campo e na análise.

Os resultados revelam extensa possibilidade para intervenções na população. O reconhecimento de subgrupos populacionais com menor cobertura de vacinação pode direcionar ações efetivas para atingir maior cobertura vacinal entre os universitários. O Brasil já usou a vacinação em escolas em outros momentos com excelentes resultados, foram realizadas campanhas vacinais contra Caxumba, Rubéola e Sarampo, com cobertura de 95%^28^. Talvez, a oferta de vacinas nas escolas, além das unidades de saúde, deslocasse a vacina do contexto de doença para o ambiente de prevenção e conhecimento que a escola consegue desenvolver melhor, explicando e integrando estudantes e pais, com o apoio de professores engajados na vacinação e treinados para dar informação.
